# 

**Published:** 1867-05

**Authors:** 


### CONTENTS.

### ORIGINAL CONTRIBUTIONS.

ART. XX. THE ACTION OF ALCOHOL UPON THE HUMAN SYSTEM. By R. L. Waltson, M.D., Paris, Ill., ........ 253

ART. XXI. REPORT ON INDIGENOUS BOTANY. By — Massey, M.D. Read before the Esculapian Society, October, 1866, ........ 270

ART. XXII. MASTITIS AND ITS TREATMENT. By Robert S. Addison, M.D., 269 Milwaukee Avenue, Chicago, ........ 279

### CORRESPONDENCE.

PATHOLOGY OF CHOLERA. By W. Duff Greene, M.D., Mount Vernon, Ill., 280

HYDROPHOBIA. Suggestions as to the Treatment of Hydrophobia. By Amos Sawyer, M.D., Hillsboro, Ill., ........ 282

### PROCEEDINGS OF SOCIETIES.

Pulaski County (Ind.) Medical Society, ........ 283
Fox River Valley Medical Association, ........ 284

### SELECTIONS.

Bloodletting Then and Now. By C. H. Spillman, M.D., of Harrodsburg, Kentucky, ........ 285

Extract from Observations upon Anæsthesia of the Cornea and Radiating Fibres of the Iris occurring without Intra-Ocular Tension. By Jos. S. Hildreth, M.D., Opthalmic and Aural Surgeon to Cook County Hospital, Chicago, Ill., ........ 290

Physiological and Therapeutical Action of Cod-Liver Oil. By A Bissell, M.D., of New York, ........ 295

Passage of an Anatomy Act in Pennsylvania ........ 300

### BOOK NOTICES.

Obstetrics; the Science and the Art. By Charles D. Meigs., M.D., lately Professor of Midwifery and Diseases of Women and Children, in Jefferson Medical College, of Philadelphia, etc., etc., etc., ........ 301
Practical Dissections. By Richard M. Hodges, M.D., ........ 301
Inhalations in the Treatment of Diseases of the Respiratory Passages, particularly as affected by the use of Atomized Fluids. By J. M. Da Costa, M.D., ........ 302
Injuries of the Spine; with an Analysis of nearly four hundred cases. By John Ashhurst, Jr., A.M., M.D., ........ 302
Code of Medical Ethics, adopted by the American Medical Association, ........ 302
The Indigestions; or Diseases of the Digestive Organs Functionally treated. By Thomas King Chambers, M.D., ........ 303

### EDITORIAL.

From the Lectures of Prof. Davis in the Medical, and Prof. Andrews in the Surgical Wards of the Mercy Hospital. [Reported for the Examiner], ........ 306
Chicago Medical Society—Annual Meeting, ........ 313
Chicago Medical College Appointments, ........ 313
Illinois State Medical Society, ........ 314
Twelve Lectures by Dr. Horatio R. Storer, ........ 314
Charitable Eye and Ear Clinic, No. 305, State St., corner VanBuren, ........ 314
Indiana State Medical Society, ........ 315
Chicago Medical Journal, ........ 315
Money Receipts from Feb. 21st to Mar. 22d, ........ 315
Mortality Report for the Month of February, ........ 316
Treatment of Acute Rheumatism, ........ 316
City and Country Population of England, ........ 317

